# EVALUATION OF STAFF TRAINING FOR DELIVERING A GROUP MOVEMENT PROGRAM TO VA NURSING HOME RESIDENTS WITH DEMENTIA

**DOI:** 10.1093/geroni/igae098.1130

**Published:** 2024-12-31

**Authors:** Matthew Miller, Delzio Molly, Margaret Chesney, Riya Jacob, Jennifer Lee, Linda Chao, Deborah Barnes, Francesca Nicosia

**Affiliations:** University of California San Francisco, San Francisco, California, United States; VA San Francisco Healthcare System, San Francisco, California, United States; University of California San Francisco, San Francisco, California, United States; San Francisco VA Healthcare System, San Francisco, California, United States; San Francisco VA Healthcare System, San Francisco, California, United States; University of California San Francisco, San Francisco, California, United States; University of California San Francisco, San Francisco, California, United States; University of California San Francisco, San Francisco, California, United States

## Abstract

Residents with dementia in VA nursing homes require complex care, but interprofessional staff rarely receive specialized training. Preventing Loss of Independence through Exercise (PLIÉ) is an evidence-based group movement program for residents with dementia that provides an opportunity for interprofessional staff to support mobility through functional movement, mindfulness, and other core principles. This study evaluated acceptability and impact of a remote program designed to increase scalability of interprofessional training for PLIÉ delivery among VA nursing home staff. The 10-week training program included weekly didactic sessions with pre-recorded videos (staff); weekly experiential sessions (staff, resident); and supervised teaching practicums (staff, residents). Satisfaction (staff, resident) and staff confidence in delivering PLIÉ were assessed using self-report questionnaires. Changes in resident fall history and physical function were examined using electronic medical records and the Minimum Data Set (MDS). Among 38 interprofessional staff enrolled across 4 sites, 29 (13 nurse, 6 recreation therapist, 10 other) attended at least 80% of the training sessions. The mean±SD staff satisfaction (N=22) was 4.5±0.7 (1: Poor, 5: Excellent) and confidence delivering PLIÉ was 6.7±0.7 (1: Cannot do, 7: Certain I can do). The mean±SD resident satisfaction (N=11) with the PLIÉ program was 3.7±1.0. The proportion of residents (N=32 residents; 31 male, 11 Black/African American) with a documented fall was lower after PLIÉ (Pre: 29%, Post: 6%, p=0.02), and attending more PLIÉ sessions was associated with physical function improvement (β:-0.83, p=0.03). These findings suggest the remote PLIÉ training program is acceptable and potentially improves resident fall rates and physical function.

